# Counterexamples to the MMP for 1-foliations in positive characteristic

**DOI:** 10.1007/s11565-024-00488-7

**Published:** 2024-02-03

**Authors:** Fabio Bernasconi

**Affiliations:** Departement Mathematik und Informatik, Universität Basel, Spiegelgasse 1, 4051 Basel, Switzerland

**Keywords:** Minimal Model Program, Foliations, Positive characteristic ground fields, Primary: 14E30, 14G17, Secondary: 14J10.

## Abstract

We show that many statements of the Minimal Model Program, including the cone theorem, the base point free theorem and the existence of Mori fibre spaces, fail for 1-foliated surface pairs $(X,\mathcal {F})$ with canonical singularities in characteristic $p>0$.

## Introduction

The Minimal Model Program (MMP) is a conjecture concerning the birational geometry of algebraic varieties, with the goal of extending the theory of minimal models from algebraic surfaces to higher dimensions. A large part of this program has been established for varieties over fields of characteristic 0 [2, 20, 25], as well as for 3-dimensional varieties in positive and mixed characteristic [4, 16, 34, 35].

In recent decades, birational geometry of foliations in characteristic 0 has emerged as a highly active research area in algebraic geometry. Starting from the work of Brunella, Mendes and McQuillan, the MMP for foliations on surfaces has been proven in [5, 27] and applied to solve the Green–Griffiths conjecture for surface with positive second Segre class [29]. More recently, significant progress has been made in extending the MMP to foliations on 3-folds, as discussed in [7, 8, 32]. Additionally, the MMP for algebraically integrable foliations in every dimension has been established in [6, 10]. Furthermore, the cone theorem for canonical Gorenstein foliations of rank 1 in every dimension has been established by Bogomolov and McQuillan [3, Corollary 4.2.1], and it has recently been extended to the log canonical setting in [9]

In light of the aforementioned developments, it is natural, from the perspective of birational geometry, to inquire whether one can extend the MMP to the case of foliations in characteristic $p>0$. The purpose of this brief note is to provide a negative response by constructing several counterexamples to the classical MMP statements for 1-foliations on surfaces in positive characteristic.

We begin with the cone theorem. Tanaka established the cone theorem for $\mathbb {Q}$-factorial projective surfaces over a perfect field of characteristic $p > 0$ in [33, Theorem 3.13]. In [31, Theorem 6.3], Spicer demonstrated the cone theorem for arbitrarily singular foliations of rank 1 on normal projective surfaces over $\mathbb {C}$. Our first result shows that the cone theorem does not hold for 1-foliations in characteristic $p > 0$.

### Theorem 1.1

For every algebraically closed field *k* of characteristic $p>0$, there exists a foliated surface pair $(X, \mathcal {F})$ such that

- $\mathcal {F}$ is a canonical 1-foliation of rank 1 on *X* such that $-K_\mathcal {F}$ is ample;
- the Mori cone ${{\,\mathrm{{\overline{NE}}}\,}}(X)$ is not finitely generated nor locally polyhedral;
- *X* does not contain any rational curves.

Another pillar of the MMP in characteristic 0 is the base point free theorem [20, Theorem 3.3]. For $\mathbb {Q}$-factorial surfaces in characteristic $p>0$, this has been proved in [33, Theorem 3.21]. A version of the basepoint free theorem has been established in the case of rank 1 foliations on surfaces in characteristic 0 by McQuillan [27, Sect. III.2] and some special cases in higher dimensions are proved ([8, Theorem 9.4] and [7, Theorem 9.2]). Here, we demonstrate that the base point free theorem does not generally hold for 1-foliations in characteristic $p > 0$.

### Theorem 1.2

For every uncountable algebraically closed field *k* of characteristic $p>0$, there exists a $\mathbb {Q}$-factorial canonical 1-foliated surface pair $(X, \mathcal {F})$ such that

1. *D* is a nef $\mathbb {Q}$-divisor on *X* such that $D-K_\mathcal {F}$ is ample, and
2. *D* is not semi-ample.

Finally, we demonstrate that Mori fiber spaces may not exist for foliated pairs with a non-pseudo-effective canonical divisor.

### Theorem 1.3

For every algebraically closed field of characteristic $p>0$, there exists a canonical 1-foliated surface pair $(X, \mathcal {F})$ such that

- $K_\mathcal {F}$ is not pseudo-effective, and
- it does not exists a $K_\mathcal {F}$-negative birational contraction $X \rightarrow Y$ such that *Y* admits a $K_\mathcal {F}$-Mori fibre space structure.

The main source of examples comes from the natural foliation $\mathcal {F}$ associated to a *p*-cyclic covering $X=Y[\root p \of {f}] \rightarrow Y$ in characteristic $p>0$, which we recall in Sect. 3.1. This construction, introduced by Ekedahl [13, page 145] and revisited by Kollár in [22], gives a counterexample to the Bogomolov–Sommese vanishing theorem in positive characteristic. It has recently been revisited by Langer to give counterexamples to Miyaoka’s generic semipositivity [24, Sect. 5.1] and by Graf to construct counterexamples to the logarithmic extension theorem for differential forms ( [14, Theorem 1.6]). Applying this construction to *p*-cyclic covering of well-chosen abelian surfaces, we construct Fano foliations which serve as the sources of counterexamples for our results in Sect. 3.2.

### Remark 1.4

The results of these notes suggest that general 1-foliations exhibit pathological behavior concerning the MMP. However, in the case of foliations defined as infinitesimal groupoids (as per [28, Definition 1.1]), McQuillan has recently established a cone theorem for absolutely $\mathbb {Q}$-Gorenstein foliations of rank 1 ( [28, Fact 5.7]) in any characteristic. This shows that Theorem 1.1 represents a phenomenon unique to such pathological 1-foliations. We also observe that our examples are 1-foliations that are not $(\infty )$-foliations in the sense of [15] as explained in [13, page 145]. This leads us to inquire whether the MMP might still hold true in the case of $(\infty )$-foliations on surfaces.

## Preliminaries

### Notations

- Throughout this article, we fix *p* to be a prime number.
- We denote by *k* an algebraically closed field.
- A *k*-variety (or simply variety) is a quasi-projective integral scheme of finite type over *k*.
- If *X* is a variety over a field *k* of characteristic $p>0$, we denote by $F :X \rightarrow X$ the *absolute Frobenius* of *X*.
- If *X* is a projective normal *k*-variety, we denote by ${{\,\mathrm{N^1}\,}}(X)$ the real numerical Néron-Severi space and by ${{\,\mathrm{N_1}\,}}(X)$ the real vector space of numerical equivalence classes of curves. The *Mori cone* ${{\,\mathrm{{\overline{NE}}}\,}}(X)$ of *X* is the closure of the real cone spanned by effective curves inside ${{\,\mathrm{N_1}\,}}(X)$.
- Given a normal *k*-variety *X*, we define its *tangent sheaf* $T_X$ as the dual of $\Omega _{X/k}^{1}$ and we denote $[ -, - ] :T_X \times T_X \rightarrow T_X$ the Lie bracket on $T_X$.

### Foliations in characteristic $p>0$

We recall the definition of a foliation on algebraic varieties.

#### Definition 2.1

Let *X* be a normal variety over *k*. A *foliation* $\mathcal {F}$ is a coherent subsheaf of $T_X$ such that

- it is saturated in $T_X$, i.e. $T_X/\mathcal {F}$ is torsion-free;
- it is closed under Lie brackets, i.e. $[\mathcal {F}, \mathcal {F}] \subset \mathcal {F}$.

We say $\mathcal {F}$ is a foliation of rank *r* if the rank of the coherent sheaf $\mathcal {F}$ is *r*. A *canonical divisor* $K_\mathcal {F}$ of a foliation $\mathcal {F}$ is a Weil divisor such that $\mathcal {O}_X(K_\mathcal {F}) \simeq \det (\mathcal {F})^{*}.$ We define $\mathcal {N}_\mathcal {F}^* = (T_X / \mathcal {F})^*$ to be the *conormal sheaf* of $\mathcal {F}$. The normal sheaf $\mathcal {N}_\mathcal {F}$ of $\mathcal {F}$ is the dual of the conormal sheaf.

From now on, we assume *k* to be a field of characteristic $p>0$. In this case, together with Lie brackets, we have an additional operation on vector fields of a variety *X*: the elevation to the *p*-th power. More precisely, we define a homomorphism of sheaves of abelian groups

$$\begin{aligned} \circ ^{[p]} :F^*T_X \rightarrow T_X, \end{aligned}$$

as follows: given an open set $U \subset X$ and for every $\mathcal {O}_X(U)$-derivation $D \in T_X(U)$, its *p*-th power $D^{[p]}$ is the composition $\underbrace{D \circ \dots \circ D}_{p\text { times}}$. Note that one can verify that $D^{[p]}$ is still a derivation by applying the Leibniz formula and the fact that $p=0$. It is easy to see that, for any $f \in \mathcal {O}_X$ and $D \in T_X$, then $(fD)^{[p]}=f^pD^{[p]}$. Note that the elevation to the *p*-th power does not respect the additive structure. In this note, we work with foliations invariant under the elevation to the *p*-th power.

#### Definition 2.2

Let *X* be a normal variety over *k*. A foliation $\mathcal {F}$ on *X* is a *1-foliation* (or a *p*-closed foliation) if the composite map

$$\begin{aligned} F :F^*\mathcal {F} \rightarrow F^*T_X \xrightarrow {\circ ^{[p]}} T_X \rightarrow TX/\mathcal {F} \end{aligned}$$

vanishes identically. Equivalently, for every open set $U \subset X$ and $D \in \mathcal {F}(U)$, the *p*-th power $D^{[p]} \in \mathcal {F}(U)$.

#### Remark 2.3

Note that the composition $F^*\mathcal {F} \rightarrow F^*T_X \rightarrow T_X \rightarrow TX/\mathcal {F}$ is $\mathcal {O}_X$-linear.

1-foliations appear naturally in the Jacobson correspondence for purely inseparable field extension of height 1 first proven in [17] (and later extended in [18]). For the formulation in terms of factorisation of the geometric Frobenius we refer to [30, Proposition 2.9] (see also [13]).

#### Theorem 2.4

(Jacobson’s correspondence) Let *X* be a normal variety over *k*. There is a one-to-one correspondence between

- 1-foliations $\mathcal {F}$ of rank r;
- Factorisation of the geometric Frobenius morphism $F_{X/k} :X \xrightarrow {f} Y \xrightarrow {g} X^{(-1)}$.

If $\mathcal {F}$ has rank r, then the degree of *f* is $\deg (f)=p^r$.

We recall the formula for the ramification formula for the canonical classes in the case of purely inseparable morphism induced by a 1-foliation.

#### Proposition 2.5

([30, Proposition2.10]) Let $\mathcal {F}$ be a 1-foliation on a normal variety *X*. Let $\pi :X \rightarrow X/\mathcal {F}$ be the purely inseparable morphism induced by $\mathcal {F}$ under the Jacobson correspondence. Then

$$\begin{aligned} \pi ^* K_{X/\mathcal {F}} - K_X = (p-1) K_{\mathcal {F}}. \end{aligned}$$

An instance where 1-foliations of rank *r* appear naturally is in presence of a separable dominant morphism $f :X \rightarrow Y$ whose generic fibre has dimension *r*. In this case, the foliation $\mathcal {F} = \text {ker}(df :T_X \rightarrow f^*T_Y)$ is a 1-foliation of rank *r*.

#### Definition 2.6

A foliation $\mathcal {F}$ on *X* is said to be *algebraically integrable* if there exists an open Zariski subset $U \subset X$ and a smooth morphism $f :U \rightarrow V$ such that $\mathcal {F}|_U = \text {ker}(df :TU \rightarrow f^*TV)$.

#### Remark 2.7

As explained in [26, Sect. 7, pp. 1362–1363] the class of 1-foliations coincides with the class of algebraically integrable foliations in characteristic $p>0$.

For smooth 1-foliations on smooth varieties, we have an analogue of Frobenius theorem.

#### Theorem 2.8

([27, Divertimento II.1.6.]) Let $\mathcal {F}$ be a smooth 1-foliation of rank 1 on a smooth variety *X* of dimension *n* (eq. $\mathcal {F} \rightarrow T_X$ is a morphism of vector bundles). Then at each point *x*, there is a choice of local parameters $x_1, \dots x_n$ around *x* such that the vector field $\partial _{x_1}$ generates $\mathcal {F}$.

Given a foliation $\mathcal {F}$ on *X* and a proper birational morphism $\pi :Y \rightarrow X$, let $\widetilde{\mathcal {F}}$ be the pull-back foliation on *Y* defined as in [12, Sect. 3.3]. In the spirit of the MMP, one can introduce the class of singularities for foliated pairs by comparing $K_\mathcal {F}$ and $K_{\widetilde{\mathcal {F}}}$. For this, it is natural to require the canonical class of the foliation to be $\mathbb {Q}$-Cartier.

#### Definition 2.9

We say $(X, \mathcal {F})$ is a *foliated pair* (resp. *1-foliated pair*) if *X* is a normal variety, $\mathcal {F}$ is a foliation (resp. 1-foliation) such that the canonical divisor $K_\mathcal {F}$ is $\mathbb {Q}$-Cartier.

As usual, we start with the definition of discrepancies. If $(X, \mathcal {F})$ is a foliated pair and $\pi :Y \rightarrow X$ is a proper birational morphism of normal varieties, We can write

$$\begin{aligned} K_{\widetilde{\mathcal {F}}} = \pi ^*K_\mathcal {F}+\sum _i a(E_i, \mathcal {F}) E_i, \end{aligned}$$

where $E_i$ runs through the $\pi$-exceptional divisors. We say that $a(E, \mathcal {F})$ is the *(foliated) discrepancy* of *E* with respect to $\mathcal {F}$.

#### Definition 2.10

We say a prime divisor $D \subset X$ is an *invariant* divisor for the foliation $\mathcal {F}$ if $\mathcal {F}|_D \rightarrow T_X|_D$ factorises through $T_D$ at the generic point of *D*. For each prime divisor *D* on *X*, we define $\epsilon (D)=0$ (resp. $\epsilon (D)=1$) if *D* is invariant (resp. not invariant) for $\mathcal {F}$.

#### Definition 2.11

A foliated pair $(X, \mathcal {F})$ has *terminal* (resp. *canonical*) singularities if $a(E, \mathcal {F}) >0$ (resp. $\ge 0$) for every exceptional prime divisor *E* over *X*. It has *klt* (resp. *log canonical*) if $a(E, \mathcal {F}) >-\epsilon (E)$ (resp. $\ge -\epsilon (E)$) for every exceptional prime divisor *E* over *X*.

#### Remark 2.12

Let $\mathcal {F}$ be a Gorenstein foliation on *X* generated by a vector field *v*. By definition, $\mathcal {F}$ is canonical if and only if for every proper birational morphism $f :Y \rightarrow X$ the rational vector field ${\widetilde{v}}$ obtained as pull-back of *v* does not vanish at the generic point of any exceptional divisor.

The next two examples should warn the reader that the notions of terminal and canonical singularities are more subtle in characteristic $p>0$ than in characteristic 0.

#### Example 2.13

Smooth 1-foliations $\mathcal {F}$ on smooth surfaces are canonical. By Theorem 2.8 we can suppose $\mathcal {F}$ is the smooth foliation generated by the vector field $\partial _x$ in $\mathbb {A}^2_{x,y}$. For every proper birational morphism $f :Y \rightarrow X$, let *v* be the rational vector field obtained as the pull-back of $\partial _x$. As *f* is birational, we have that $f^*(dx)(v)=1$. As $f^*(dx)$ is a regular 1-form, we conclude that *v* cannot acquire zeroes at the generic point of any exceptional divisors, showing that $\mathcal {F}$ has canonical singularities by Remark 2.12.

However, it is important to note that $\mathcal {F}$ is not terminal in characteristic $p > 0$, as pointed out in [27, Counterexample I.2.11]. This contrasts with the case of terminal excellent surfaces, which are shown to be regular in [21, Theorem 2.29]. To show that $(X, \mathcal {F})$ is not terminal, it is sufficient to perform the weighted blow-up $\pi :Y {:}{=} \text {Bl}_{(1,p)}\mathbb {A}^2 \rightarrow \mathbb {A}^2$ with weight (1, *p*) at the origin. In a local chart of *Y* containing the generic point of the exceptional divisor *E* of $\pi$, the morphism $\pi$ is described by $(s,t) \mapsto (s, s^p t).$ As *dy* is a local section of the conormal bundle $\mathcal {N}^*_{\mathbb {A}^2}$ together with the formulas $\pi ^*dy=s^pdt, \text { and }\pi ^*(dx \wedge dy)=s^p ds \wedge dt$ we conclude that

$$\begin{aligned} K_{Y} = \pi ^{*}K_X+pE \text { and } \mathcal {N}^*_{Y} = \pi ^* \mathcal {N}^*_{\mathbb {A}^2}+pE. \end{aligned}$$

Thus $K_{\widetilde{\mathcal {F}}} = \pi ^* K_{\mathcal {F}}$, concluding that $\mathcal {F}$ does not have terminal singularities.

If $(X,\mathcal {F})$ is a canonical surface foliated pair in characteristic 0, then *X* has log canonical singularities [27, Fact I.2.4 and Theorem III.3.2], and this has been extended to rank 1 foliations on 3-folds in [8, Theorem 1.5]. The following shows that this is no longer true for canonical 1-foliations in positive characteristic.

#### Example 2.14

Let $X:= \left\{ z^p-f(x,y)=0 \right\} \subset \mathbb {A}^3_{x,y,z}$ be a normal hypersurface singularity. In characteristic $p>0$, *X* is an invariant divisor on $\mathbb {A}^3$ for the 1-foliation described by the vector field $\partial _z$ on $\mathbb {A}^3$. Therefore we can restrict $\partial _z$ on *X*, which naturally defines an induced 1-foliation $\mathcal {F}$ on *X*. Note that in characteristic 0, *X* is not invariant for $\partial _z$.

We claim that $\mathcal {F}$ has canonical singularities. Let $f :Y \rightarrow X$ be a proper birational morphism and let *v* be the rational vector field on *Y* obtained as the pull-back $\partial _z$. As $f^*(dz)$ is a regular 1-form on *Y* and $f^*(dz)(v)=1$, we conclude that *v* does not acquire zeroes along the exceptional divisor and $\mathcal {F}$ has canonical singularities by Remark 2.12. Note that the singularities of *X* can be easily worse than log canonical by choosing appropriate *f*.

## Proof of the main theorems

In this section, we show the main counterexamples to the MMP for 1-foliations of this note. We start with the review the construction of *p*-cyclic covering and their properties in characteristic $p>0$ following [22].

### 1-Foliations from *p*-cyclic covering

We fix *k* to be an algebraically closed field of characteristic $p>0$ and let *Y* be a normal projective variety over *k* of dimension *n* endowed with an invertible sheaf *L*.

Let $\pi :\mathbb {L}:={{\,\mathrm{{Spec}}\,}}_Y \bigoplus _{m \ge 0} L^{-\otimes m} \rightarrow Y$ be the associated line bundle to *L* together with its tautological section $y_L \in H^0(\mathbb {L}, \pi ^*L)$. Given $s \in H^0(Y, L^{\otimes d})$ a non-zero global section for some $d>0$, we can construct the *d*-th cyclic cover $\pi :X \rightarrow Y$ along *s* defined by the vanishing of the global section $(y_L^{d}-\pi ^*s) \in H^0(\mathbb {L}, \pi ^*L^{\otimes d})$. Sometimes we denote *X* by $Y[\root d \of {s}]$. If $U \subset Y$ is an open subset such that $L|_{U} \simeq \mathcal {O}_U$, we have an isomorphism $X \simeq {{\,\mathrm{{Spec}}\,}}_U \mathcal {O}_U[y]/(y^d-f)$ over *U* for some $f \in \mathcal {O}_U$. In particular, if *Y* is Cohen–Macaulay (resp. Gorenstein, locally complete intersection), then so is *X*. We recall a few basic sequences of differentials we will need to construct a 1-foliation on *X*.

#### Lemma 3.1

(cf. [22, Lemma 9]) We have the following short exact sequences:

- On $\mathbb {L}$, we have $0 \rightarrow \pi ^*\Omega _Y^1 \rightarrow \Omega ^1_\mathbb {L} \rightarrow \pi ^*L^{-1} \rightarrow 0.$
- On *X*, we have $\pi ^*L^{-\otimes d} \xrightarrow {d_X} \Omega ^1_\mathbb {L}|_X \rightarrow \Omega ^1_X \rightarrow 0,$ where, on the smooth locus of *X*, $d_X$ is locally described by
  $$\begin{aligned} \left( -\frac{\partial s}{\partial x_1} dx_1, \dots , -\frac{\partial s}{\partial x_n} dx_n, dy^{d-1}dy \right) . \end{aligned}$$

We now specialize to the case where *p* divides *d*.

#### Lemma 3.2

If $p \mid d$, we have the following exact sequence on *X*:

$$\begin{aligned} 0 \rightarrow {{\,\mathrm{{coker}}\,}}\left( \pi ^*L^{-\otimes d} \rightarrow \pi ^*\Omega ^1_Y \right) \rightarrow \Omega _X^1 \rightarrow \pi ^*{L^{-1}} \rightarrow 0. \end{aligned}$$

Let *Y* be a smooth variety together with a very ample line bundle *L* and a global section $s \in H^0(Y, L^{\otimes p})$. On $X {:}{=} Y[\root p \of {s}]$, the homomorphism $\Omega _X^1 \rightarrow \pi ^*L^{-1}$ constructed in Lemma 3.2 defines a foliation $\mathcal {F}$ of rank 1 on *X* whose canonical bundle $K_{\mathcal {F}}=\pi ^*{L}^{-1}$ is anti-ample. We say $\mathcal {F}$ is the *foliation associated to the p-th cyclic cover* defined by the pair (*L*, *s*).

#### Lemma 3.3

The foliation $\mathcal {F}$ is *p*-closed.

#### Proof

In analytic coordinates, *X* is described by $\big \{ z^p-f=0 \big \}$ where $f \in k[x_1, \dots , x_n]$. The foliation $\mathcal {F}$ is generated by the vector field $\partial _z$, which is obviously *p*-closed. $\square$

In general, *X* is not smooth even if ($s=0$) is a smooth divisor. Nevertheless, the singularities appearing on *X* can be controlled by choosing *s* generically enough as explained in [22]. When $p \mid d$, then $L^{\otimes d}$ has a natural operator:

$$\begin{aligned} d :L^{\otimes d} \rightarrow L^{\otimes d} \otimes \Omega ^1_Y, \end{aligned}$$

which is defined as follows (see [22, Definition-Lemma 13]): choose a local generator $\tau$ of *L* and for each section $s =f \tau ^{\otimes d}$ of $L^{\otimes d}$ we define $d(s) {:}{=} df \otimes \tau ^{\otimes d}$. This operator is indipendent of the choice of a generator $\tau$. If we choose a local system of parameters $x_1, \dots , x_n$ near a point *x*, the matrix

$$\begin{aligned} H(s) {:}{=} \left( \frac{\partial ^2 f}{\partial x_i \partial x_j} \right) _{i, j} \end{aligned}$$

is called the Hessian of *s* and the rank of *H*(*s*)(*x*) is independent of the choices made.

#### Definition 3.4

We say that $s \in H^0(X, L^{\otimes d})$ has a *critical point* at *x* if $ds(x)=0$. We say a critical point *x* is *non-degenerate* if the rank of Hessian *H*(*s*)(*x*) is equal to the dimension of *X*.

We recall the following local result which relates the zeros of *ds* with the singularities of $Y[\root k \of {s}]$ and describes the singularities of the cover if the points are non-degenerate.

#### Lemma 3.5

([22, 20]) If $p \mid d$, the hypersurface ($y^d-s(x_1, \dots , x_n)=0$) is singular at the point $(y, x_1, \dots , x_n)$ if and only if $(x_1, \dots , x_n)$ is a critical point of *s*. Moreover, *s* is non-degenerate if and only if we can write, up to a change of coordinates,

$$\begin{aligned} s(x_1, \dots , x_n)= {\left\{ \begin{array}{ll} x_1x_2+x_3x_4+ \dots +x_{n-1}x_n +f_3(x), &{} \text{ if } n \text{ even } \\ x_1^2+x_2x_3+ \dots + x_{n-1}x_n+f_3(x), &{} \text{ if } n \text{ odd } \end{array}\right. } \end{aligned}$$

where $$f_3 \in (x_1, \dots , x_n)^3$$.

### Counterexamples

We recall how the numerical spaces of cycles change under purely inseparable morphisms.

#### Lemma 3.6

Let $f :X \rightarrow Y$ be a purely inseparable morphism of projective normal varieties over *k*. Then $f_* :{{\,\mathrm{N_1}\,}}(X) \rightarrow {{\,\mathrm{N_1}\,}}(Y)$ is an isomorphism. Under this isomorphism, ${{\,\mathrm{{\overline{NE}}}\,}}(X)$ is mapped onto ${{\,\mathrm{{\overline{NE}}}\,}}(Y)$.

#### Proof

The proof is dual to the one in [19, Lemma 1.4]. As *f* is purely inseparable, there exists $l \ge 0$ and a factorisation of the *l*-th geometric Frobenius morphism $F^{l}_{X/k} :X \xrightarrow {f} Y \rightarrow X^{(-l)}$. Given a curve class $[C] \subset {{\,\mathrm{N_1}\,}}(X)$, we have $(F^l_{X/k})_* [C]=p^l [C]$ and this concludes the proof of the lemma. $\square$

We are now ready to introduce the main example of this note by taking *p*-cyclic covering of abelian surfaces. A similar construction has been used in [24, Sect. 5.1] to construct counterexamples to Miyaoka’s semipositivity theorems in positive characteristic.

#### Proposition 3.7

Let $Y:=A$ be an abelian surface equipped with a very ample line bundle *L* on *Y*, and let $s \in H^0(X,L^{\otimes p})$ be a general section. Let $X:=Y[\root p \of {s}]$ be the *p*-th cyclic covering together with the induced foliation $\mathcal {F}$. Then

- *X* has canonical singularities of type $A_{p-1}$,
- $(X, \mathcal {F})$ is a canonical 1-foliated pair,
- $-K_\mathcal {F}$ is ample (i.e. $\mathcal {F}$ is a Fano foliation with canonical singularities).

#### Proof

By [22, Proposition 18], we can choose *s* to have only non-degenerate critical points. The fact that $\mathcal {F}$ is a 1-foliation follows from Lemma 3.3 and the Fano condition follows from $K_\mathcal {F}=\pi ^{*}L^{-1}$. On a smooth big open set *U* of *Y* along which *s* has no critical points, the foliation $\mathcal {F}$ is generated by the vector field $\partial _z$, thus concluding that $\mathcal {F}$ is a smooth foliation on $p^{-1}(U)$, and so canonical by Example 2.13. Around a non-degenerate critical point, we have in analytic coordinates that *X* is described by $\left\{ z^p-xy=0 \right\}$ by Lemma 3.5 and the foliation $\mathcal {F}$ is thus canonical by Example . $\square$

#### Proof of Theorem 1.1

Let *Y* be an abelian surface of Picard rank $\rho (Y) \ge 3$, together with an ample line bundle *L* and consider the foliated pair $(X, \mathcal {F})$, where $\mathcal {F}$ is the Fano 1-foliation of Proposition 3.7. Note there are no rational curves on *X* since *X* admits a finite morphism to an abelian surface. Moreover, the Mori cone ${{\,\mathrm{{\overline{NE}}}\,}}(X)$ is isomorphic to ${{\,\mathrm{{\overline{NE}}}\,}}(Y)$ by Lemma 3.6 and this is not finitely generated nor locally polyhedral by [11, 6.3]. $\square$

#### Remark 3.8

A bend-and-break result for 1-closed foliation has been proven by Langer ( [23, Theorem 2.1]). In order to produce rational curves one needs to require the following lower bound on the foliated canonical class:

$$\begin{aligned} -K_\mathcal {F} \cdot C > \frac{K_X \cdot C}{p-1}. \end{aligned}$$

Note that our example shows that the bound of Langer for the bend-and-break is optimal. Indeed, the ramification formula Proposition 2.5 implies

$$\begin{aligned} -K_{\mathcal {F}} =\pi ^*L= \frac{K_X-\pi ^*K_Y}{p-1}=\frac{K_X}{p-1}. \end{aligned}$$

Note that, even in Langer’s range, we still do not know if the rational curves produced are tangent to the foliation.

We show the base point free theorem fails for the case of 1-foliations.

#### Proof of Theorem 1.2

Let *k* be an uncountable algebraically closed field of characteristic $p>0$. Let *Y* be an abelian surface over *k*, together with an ample line bundle *L* and consider the foliated pair $(X, \mathcal {F})$, where $\mathcal {F}$ is the Fano 1-foliation of Proposition 3.7. Let $D \in {{\,\textrm{Pic}\,}}^0(A)$ be a nef divisor which is not semi-ample (equiv. not-torsion, whose existence is guaranteed by the hypothesis on *k*). By [19, Lemma 1.4], on *X* the divisor $\pi ^*D$ is nef but not semi-ample. Note that $\pi ^*D - K_\mathcal {F}\equiv -K_\mathcal {F}$ is ample, thus concluding. $\square$

We show that Mori fibre spaces do not always exist.

#### Proof of Theorem 1.3

Let *A* be a simple abelian surface of Picard rank 2. By [1, Lemma 1.1 and Proposition 1.2] (the characteristic assumption is not needed in the proof), the two extremal rays of the nef cone are irrational. Let $X:=A[\root p \of {s}]$ be a *p*-cyclic cover with the foliation $\mathcal {F}$ of Proposition 3.7. In this case, by Lemma 3.6 the two extremal rays of the nef cone of *X* are irrational and thus *X* does not admit any Mori fibre space structure. $\square$

We conclude by noting the BAB conjecture fails for canonical Fano 1-foliations.

#### Example 3.9

(Non-boundedness of Fano) The foliated pairs $(X, \mathcal {F})$ obtained by taking larger and larger powers of *L* in Proposition 3.7 show that canonical Fano 1-foliations on surfaces in characteristic $p>0$ are not foliated bounded as there is no upper bound to the self-intersection numbers $K_\mathcal {F}^2$.
